# Developing product quality standards for wheelchairs used in less-resourced environments

**DOI:** 10.4102/ajod.v6i0.288

**Published:** 2017-09-08

**Authors:** Anand Mhatre, Daniel Martin, Matt McCambridge, Norman Reese, Mark Sullivan, Don Schoendorfer, Eric Wunderlich, Chris Rushman, Dave Mahilo, Jon Pearlman

**Affiliations:** 1 Human Engineering Research Laboratories, Department of Veterans Affairs, United States; 2 Department of Rehabilitation Science and Technology, University of Pittsburgh, United States; 3 International Society of Wheelchair Professionals, University of Pittsburgh, United States

## Abstract

**Background:**

Premature failures of wheelchairs in less-resourced environments (LREs) may be because of shortcomings in product regulation and quality standards. The standards published by the International Organization for Standardization (ISO) specify wheelchair tests for durability, safety and performance, but their applicability to products used in the rugged conditions of LREs is unclear. Because of this, wheelchair-related guidelines published by the World Health Organization recommended developing more rigorous durability tests for wheelchairs.

**Objectives:**

This study was performed to identify the additional tests needed for LREs.

**Methods:**

First, a literature review of the development of ISO test standards, wheelchair standards testing studies and wheelchair evaluations in LREs was performed. Second, expert advice from members of the Standards Working Group of the International Society of Wheelchair Professionals (ISWP) was compiled and reviewed.

**Results:**

A total of 35 articles were included in the literature review. Participation from LREs was not observed in the ISO standards development. As per wheelchair testing study evidence, wheelchair models delivered in LREs did not meet the minimum standards requirement. Multiple part failures and repairs were observed with reviewed field evaluation studies. ISWP experts noted that several testing factors responsible for premature failures with wheelchair parts are not included in the standards and accordingly provided advice for additional test development.

**Conclusion:**

The study findings indicate the need to develop a wide range of tests, with specific tests for measuring corrosion resistance of the entire wheelchair, rolling resistance of castors and rear wheels, and durability of whole wheelchair and castor assemblies.

## Introduction

There remains a vast need for quality wheelchairs around the world. The World Health Organization (WHO) estimates that 10% of people with disabilities (around 111 million) require a wheelchair and only about 5% – 15% have access to an appropriate one, suggesting that the unmet need is approximately 95 million wheelchairs (Borg & Khasnabis 2008; Handicap International 2013; World Health Organization 2011). To address this need and improve the quality of life of wheelchair users and others with disabilities, international organisations are promoting improved accessibility to appropriate technology. For example, the United Nations Convention on the Rights of Persons with Disabilities (UN-CRPD), which has been ratified by 156 countries, specifically mentions the importance of assistive technologies (ATs) in eight of its Articles (4, 9, 20, 21, 24, 26, 29 and 32) (United Nations 2006). Article 20 of the UN-CRPD which focuses on personal mobility indicates that state parties must facilitate personal mobility for people with disabilities that is affordable, high quality and includes relevant training. Although there is widespread ratification of the UN-CRPD, progress on its implementation is hampered by lack of understanding of disability issues, provision of quality services, training, coordination and guidance to support member states to implement changes (International Disability Alliance 2010).

Many initiatives are underway to address wheelchair affordability, quality and relevant training. To accelerate the implementation of UN-CRPD initiatives, the UN partnered with WHO in 2013 and initiated a programme called the Global Cooperation on Assistive Technology (GATE) (World Health Organization 2014). As a part of this programme, WHO recently published a Priority Assistive Products List which among other includes both manual and attendant-propelled wheelchairs with and without postural support options (World Health Organization 2016). The WHO, furthermore, has published guidelines for provision of manual wheelchairs in less-resourced environments (LREs) and developed wheelchair service training packages in partnership with the United States Agency for International Development (USAID) (Borg & Khasnabis 2008; World Health Organization 2012, 2013). The International Society of Wheelchair Professionals (ISWP) was formed in 2015 with a seed grant from USAID to the University of Pittsburgh (International Society of Wheelchair Professionals 2015). The ISWPs’ mission is to professionalise the wheelchair sector by promoting standardisation of wheelchair services, coordinating wheelchair activities and raising awareness of the need for proper wheelchair services around the world. While many international efforts are in progress, it has been noted that provision of high-quality products is challenging because of lack of controls (regulations), adoption of product and service provision standards, funding, disability inclusion in policies, trained personnel and awareness in LREs (Borg, Lindström & Larsson 2011; Marasinghe, Lapitan & Ross 2015; Oderud 2014; Sheldon & Jacobs 2006; Visagie, Duffield & Unger 2015a; Visagie, Scheffler & Schneider 2013).

A key document outlining mobility needs in LREs are the WHO guidelines, which specify best practices for wheelchair design, production and supply, with a focus on increasing the quality of products (Borg & Khasnabis 2008). The guidelines emphasise the consideration of the unique environments of LREs when designing for strength and durability. Outdoor environments in LREs often include unpaved and soft surfaces, muddy roads, potholes, high curbs, gravel, sand, water, steep inclines and inaccessible buildings and public spaces (Borg & Khasnabis 2008; Chakwizira et al. 2010; Constantine, Hingley & Howitt 2006; Glumac et al. 2009; Hotchkiss 1985; Kim & Mulholland 1999; Rispin & Wee 2015; Sheldon & Jacobs 2006). Manoeuvring over rocky surfaces and obstacles exposes wheelchairs to heavy shocks and persistent vibrations. Varying seasonal conditions, elevated temperatures and high humidity (Borg & Khasnabis 2008) foster increased corrosion, ageing and wear. Such unique conditions place additional requirements on wheelchair durability which can cause premature failures if the product quality is poor (Borg & Khasnabis 2008; Marasinghe et al. 2015; Sheldon & Jacobs 2006). Failures in the community because of product design-environment mismatch can cause consequences such as accidents, frequent repairs and breakdowns (Borg & Khasnabis 2008; Cooper et al. 2004; Fitzgerald et al. 2005; Gaal et al. 1997; Kim & Mulholland 1999; Toro et al. 2012; Visagie et al. 2013, 2015a).

User behaviours are also different in LREs compared to those in resourced environments (REs), which should be considered during wheelchair design (Borg & Khasnabis 2008; Glumac et al. 2009; Marasinghe et al. 2015; Mulholland et al. 1998). For instance, wheelchairs must withstand the stresses caused by rough handling, as they are tossed on and off the roof of a bus (Borg & Khasnabis 2008). Furthermore, they need to be light and compact enough to be agile and easily portable (Hotchkiss 1985). Additionally, users often leave their wheelchairs outside exposed to the weather, or use them as shower chairs (Borg & Khasnabis 2008; Pearlman et al. 2008). Users also frequently transport goods on the push handles, seats, footrests or other parts of the wheelchair as well as carry passengers on armrests or footrests (Borg & Khasnabis 2008). Thus, the diverse functional requirements for wheelchairs impose greater durability requirements on the designs.

**FIGURE 1:**
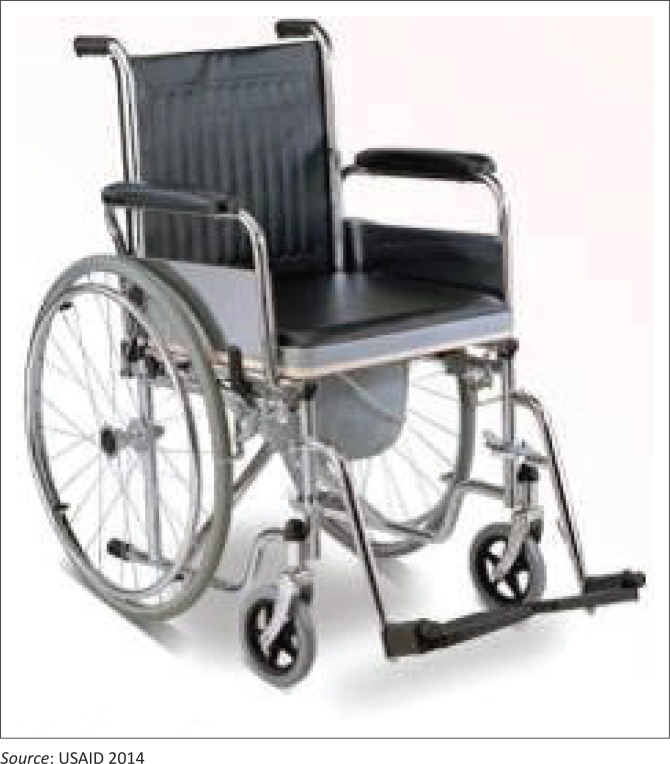
Hospital style wheelchair.

Quality of designs provided in LRE contexts varies based on the service delivery and funding methods (Oderud 2014; Pearlman et al. 2006; Visagie et al. 2015a). Donated, refurbished and locally produced wheelchair models are often hospital style (see Figure 1). These designs are not appropriate for outdoor use as they are based on designs for indoor and institutional use (Constantine et al. 2006; Glumac et al. 2009; Lysack et al. 1999; Oderud 2014; Pearlman et al. 2006; Rispin & Wee 2015; Sheldon & Jacobs 2006; Visagie et al. 2015b). In LREs, quality is often traded for cost savings as some designs include plastic wheels and cushions which are not durable enough, while some lack features like folding frame and essential parts such as parking brakes, push rims, resilient castors, etc. (Constantine et al. 2006; Hof, Hotchkiss & Pfaelzer 1993; Oderud 2014; Rispin & Wee 2015; Rispin et al. 2013) which makes the product inappropriate for use. As an example, more than 75% of users (*n* = 94) were found to be dissatisfied with the durability and weight of unsuitable products that were provided in Zimbabwe (Visagie et al. 2015b). Anecdotal reports mention that donated wheelchairs often last no more than 3–6 months (Constantine et al. 2006; Sheldon & Jacobs 2006; Oderud 2014). Lack of context-appropriate designs and high-quality products can lead to decreased functional status, secondary health complications, breakdowns and repairs (Oderud 2014; Visagie et al. 2015a, 2015b).

Regular servicing and maintenance are necessary for reducing breakdowns, repairs, occurrence of adverse events (e.g. accidents) and improving reliability (Chen et al. 2011; Gaal et al. 1997; Hansen, Tresse & Gunnarsson 2004; Toro et al. 2012). WHO guidelines recommend conducting user training in basic wheelchair repair and regular maintenance by wheelchair service personnel (Borg & Khasnabis 2008). However, lack of wheelchair service professionals and limited awareness of best service delivery practices make user training difficult. Lack of necessary resources (materials, availability of spare parts, tools and equipment and workshop facilities) for repairs and skilled technical labour create challenges for maintenance (Armstrong, Reisinger & Smith 2007; Borg & Khasnabis 2008; Hof et al. 1993; Oderud 2014; Sheldon & Jacobs 2006; Visagie et al. 2013, 2015b). Furthermore, if an imported or donated wheelchair breaks down, it is difficult to find replacement parts and expensive to buy or import them (Constantine et al. 2006; Kim & Mulholland 1999; Sheldon & Jacobs 2006). As a result, breakdowns are not quickly addressed (Borg et al. 2011; Pearlman et al. 2008) and failures and unavailable repairs can make loss of mobility long term, especially because users in LREs do not have backup wheelchairs (Hotchkiss 1985). This, in turn, has multidimensional consequences for the user, including reduced satisfaction and increased likelihood of device abandonment (Fitzgerald et al. 2005; Phillips & Zhao 1993). Thus, the lack of repair options in LREs makes the need for durable chairs even greater.

The aforementioned problems with product quality and their corresponding impact on the user’s quality of life were highlighted during a consensus conference held in 2006 by several experts and stakeholders involved in wheelchair provision in LREs (Sheldon & Jacobs 2006). The outcome of this conference was the development of WHO guidelines that called for testing of wheelchairs delivered in LREs. The WHO guidelines further advocated using international wheelchair testing standards developed by the International Organization for Standardization (ISO) as a basis to develop and adopt national standards in LREs. The ISO 7176 series includes wheelchair standards that are intended to apply universally to all contexts, and many national standards committees have adopted ISO 7176 (Borg & Khasnabis 2008). For instance, in United States, the Rehabilitation Engineering Society of North America (RESNA) Standards Committee under American National Standards Institute (ANSI) approval has led the development of ANSI/RESNA standards which are mostly consistent with ISO 7176 (Cooper, Boninger & Rentschler 1999; Rehabilitation Engineering Society of North America 2009). The ISO 7176 has been adopted in Great Britain, South Africa, China, Australia and New Zealand as well. These standards address safety, durability, manoeuvrability and transport (International Organization for Standardization 2014). Further recommendation by the WHO guidelines to improve product quality was to include additional tests to evaluate wheelchairs for environmental, user and resource conditions experienced in LREs (Borg & Khasnabis 2008). With WHO recommendations in mind, the authors undertook this study to identify exactly which additional tests need to be developed.

## Methods

The additional tests suggested in this article were based on a literature review of wheelchair standards development, wheelchair standards testing and wheelchair field evaluations in LREs as well as advice from a group of experts. A detailed description of the methods is described below.

### Literature review methods

A literature search was conducted on scientific and medical databases from the earliest time permitted electronically using PubMed, CIRRIE, EBSCO Host and Scopus. Keywords used for searching titles (and title or abstract for PubMed) in alphabetical order were: wheelchair + ANSI/RESNA, assessment, comparison, environment, evaluation, ISO, performance, review, standards and testing. There was no limitation placed on the year of publication. Duplicates were removed and titles of the selected articles were screened by the author and assisting researcher and saved for further screening. Articles were then retrieved using the University of Pittsburgh library. Further review of articles based on abstracts was carried out by the author and the researcher. If any article was deemed relevant to the topics of interest by only one reviewer as per the abstract, then both reviewers read through the article to determine its relevance. Studies on motorised wheelchairs, scooters and manual suspension wheelchairs were not taken into account as the available wheelchairs used in LREs are mostly manual (Hof et al. 1993). The articles that were deemed relevant were read entirely and reviewed by the author and other researcher for inclusion in this literature review. References found from screened articles were searched using PubMed and Google Scholar or physically retrieved. Included articles were categorised into the three categories: (1) ISO standards development, (2) wheelchair testing with ISO standards and (3) field evaluation studies reporting wheelchair failures in the community. Studies conducted in REs were excluded from the third category.

Data collection and analysis were performed by the primary author. The articles related to ISO standards were evaluated for understanding whether LRE conditions were considered during the test method development process. Extracted elements from studies on ISO wheelchair testing included wheelchair sample size, ISO durability testing results and part failures. For articles related to wheelchair evaluation in LRE communities, information was retrieved on study design, wheelchair ISO qualification, maintenance status and field failures.

### Expert advice

Advice on additional test development was sought from nine members of the ISWP Standards Working Group (ISWP-SWG). This expert group is composed of wheelchair manufacturers, designers and providers from charitable organisations and field experts with work experiences in LREs. All experts were familiar with ISO 7176 test methods. Information on failures in LREs and test development was collected through biweekly group discussions through Web conferencing via Adobe Connect (Adobe Systems Incorporated 2016). ISWP-SWG members provided pictures of broken and inoperable parts that they had collected through their work to demonstrate the types of failures common in LREs. Group discussions were centred around these failures that are not predicted by ISO 7176 tests. The failure photos were instrumental in gaining consensus about the common failures and making suggestions for the additional tests needed. Votes were taken within the group to nominate parts for testing consideration.

### Additional test method identification

A systematic process was used to generate a prioritised list of the new tests recommended from this work. First, a product testing matrix was generated that includes a column listing the failures common in LREs that were identified through the literature review and by the members of the ISWP-SWG. Test conditions responsible for failures were noted. Second, experts determined whether the test conditions are already included in ISO 7176. Third, if a need for additional testing was identified, an effort was made to leverage existing test methods from relevant ISO standards, American Society for Testing and Materials (ASTM) standards and United States Military Standards (MIL-SPEC). If it was determined that a suitable test method did not already exist, members from the ISWP-SWG made suggestions for new test methods. Voting was carried out in the group to select test methods to be developed by ISWP.

## Results

**FIGURE 2:**
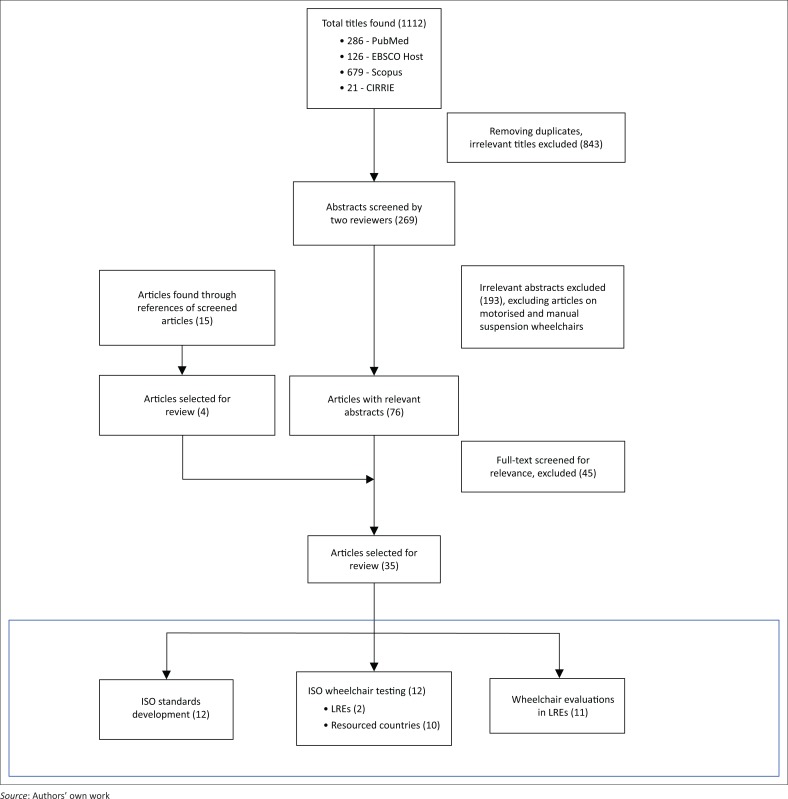
Flowchart of article selection process for review.

The flow chart outlining the selection process of articles is shown in Figure 2. Of the 1112 citations retrieved and 15 citations found through references of screened articles, 35 articles met the inclusion criteria and were categorised and analysed further.

### International Organization for Standardization standards development

International Organization for Standardization work commenced in the early 1980s with participation from UK, Sweden, Germany, France, Denmark, United States, Canada, Austria and Japan (Cooper et al. 1996a; McLaurin 1986; Staros 1981), but no LREs were involved. In the 1990s, the ISO committee developed and published wheelchair standards (Cooper et al. 1996a; Hobson 1999) and expanded participation (International Organization for Standardization 2014). Currently, there are 24 countries participating in the ISO standards committee (plus 11 observing countries) including Brazil, China and India which are considered less-resourced countries. Working groups under this subcommittee typically meet twice a year for developing new standards and revising existing standards (Cooper et al. 1996a). There are now 34 standards published by the committee with expanded categories that include power wheelchairs, scooters and stair-climbing devices. Standards specify disclosure requirements for testing and methods of measurement for: static stability (§1), dynamic stability (§2), brake effectiveness (§3), energy consumption (§4), wheelchair and seat dimensions (§5), maximum speed, acceleration and deceleration (§6), determination of seating and wheel dimensions (§7), static, impact and fatigue strength testing (§8), climatic testing (§9), obstacle climbing ability (§10), test dummy specifications (§11), power and control system (§14), flammability requirements (§16), electromagnetic compatibility (§21), setup procedures (§22) and vocabulary (§26) (International Organization for Standardization 2014). In all, wheelchair standards tests consist of durability, safety and performance tests along with measurement and reporting of wheelchair dimensions and characteristics. Some test procedures allow for comparison between wheelchair safety and performance, while certain tests need the wheelchair to pass minimum requirements (Cooper et al. 1996a; Hobson 1999; International Organization for Standardization 2014).

**FIGURE 3:**
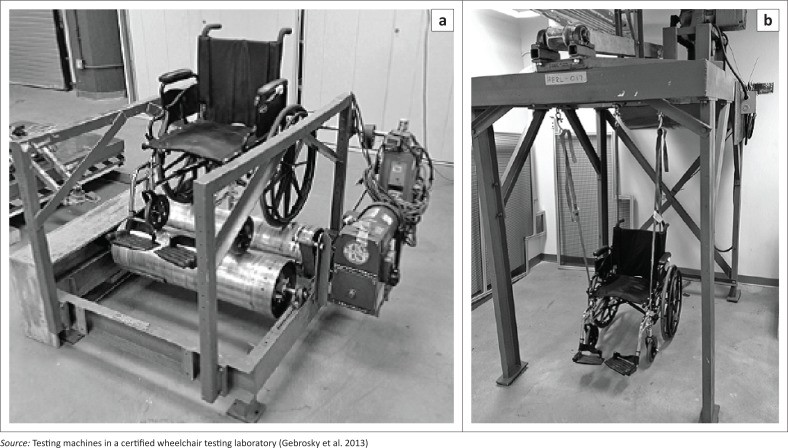
Multidrum test (left) and curb-drop test (right) without test dummies.

The ISO 7176-8 suite of durability tests includes tests for strength, impact and fatigue which primarily assess a wheelchair’s quality. Strength tests require static loading of armrests, footrests, handgrips, push handles and tipping levers. Impact tests are conducted with a test pendulum on backrests, hand rims, footrests and castors. Fatigue tests consist of a multidrum test (MDT) of 200 000 cycles and a curb-drop test (CDT) of 6666 cycles (see Figure 3). Failures of the MDT and CDT are classified into three classes: Class I and Class II failures are because of maintenance issues and can be fixed by a user or dealer, while Class III failures are caused by structural damage and require a major repair or part replacement (Cooper 1998). A Class III failure indicates failure of the test.

### Wheelchair testing with International Organization for Standardization standards

**FIGURE 4:**
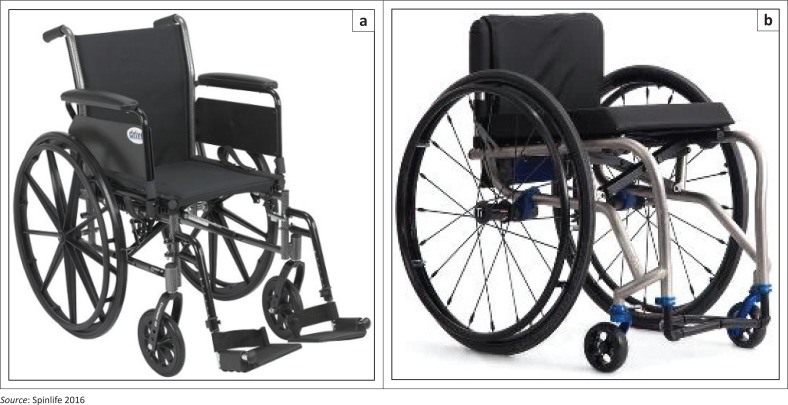
Lightweight wheelchair (left) and ultra-lightweight wheelchair (right).

The literature review on wheelchair testing with ISO standards focused on 12 articles (Cooper et al. 1996b, 1997, 1999; Cooper, Stewart & VanSickle 1994; Fitzgerald et al. 2001; Gebrosky et al. 2013; Liu et al. 2008, 2010; Rentschler et al. 2001; Toro et al. 2013; Wang et al. 2010; Zipfel et al. 2007) that deal with laboratory testing of different wheelchair designs. Included were hospital style (HWC), lightweight (LWC) and ultra-lightweight (UWC) wheelchairs (see Figure 4). Wheelchair models were in new condition and were already available on the market. Information regarding their prior ISO testing was not available. Some testing studies referred to ANSI/RESNA standards. Table 1 presents study results from ISO section 8 tests and lists the observed failures. Among different designs, UWCs were found to be more durable and cost-effective compared to LWCs and HWCs except in the most recent study by Liu et al. (2010). UWCs experienced higher Class I failures that could be repaired by users, whereas HWCs had greater Class III failures (Fitzgerald et al. 2001).

**TABLE 1:** Findings from the International Organization for Standardization standard testing studies of manual wheelchairs.

Author and year	Wheelchair samples	Test results and critical failures
**ISO testing of manual wheelchairs (no data available on design type and manufacturers)**		
Cooper et al. (1994)	Nine manual wheelchairs	All wheelchairs failed on MDT. Failures observed with castor spindle, bearings and alignment. Bent cross braces were found. Splaying and toe-outs observed in rear wheels.
Rentschler et al. (2001)	46 manual wheelchairs	Twenty-seven of 46 wheelchairs failed the MDT and CDT tests. Twenty-eight of 38 wheelchairs tested until failure incurred frame failures.
Wang et al. (2010)	154 manual wheelchairs	Seventy-five of 154 wheelchairs failed the MDT and CDT tests. No evidence on type of failures was included.
**ISO testing of wheelchairs produced and used in LREs**		
Toro et al. (2013)	Two HWC models	Both wheelchairs failed MDT. Failures noted were wheel coming off axle, flat pneumatic insert and tyre, right hub failure, castor tyre wear out and castor fork crack.
Zipfel et al. (2007)	One HWC	Wheelchair failed on MDT. Cross brace failure occurred.
**ISO testing of wheelchairs used in resourced countries**		
Fitzgerald et al. (2001)	Sixty-one manual wheelchair models from four manufacturers: 25 HWCs, 22 UWCs and 14 LWCs	Eighty-three per cent of the HWCs, 61% of the LWCs and 24% of the UWCs failed MDT. Twenty-one Class I failures, 29 Class II failures and 45 Class III failures were noted. Castor assembly and frame failures were found.
Cooper et al. (1996b)	Six HWCs and nine UWCs	All HWCs failed the MDT. One of nine UWCs failed on CDT. Failures with footrest weld, castor spindles, side frame, cross braces and castor spokes were reported.
Cooper et al. (1997)	Three samples of three LWC models	Eight of nine LWCs failed MDT and CDT tests. Several side frame failures occurred in weld areas, one castor spindle failure and one cross brace failure.
Gebrosky et al. (2013)	Three samples of three LWC models	All wheelchairs passed the strength tests. Seven of nine LWCs failed the MDT and CDT tests. Several frame failures were observed.
Liu et al. (2008)	Three samples of four aluminium rigid UWC models	All wheelchairs passed impact strength tests and brake fatigue tests. Five of 12 chairs failed MDT and CDT tests.
Cooper et al. (1999)	Three samples of four UWC models	One of 12 UWCs failed MDT and CDT tests. Castor stem failures, weld, rear wheel bearing and frame failure were noted.
Liu et al. (2010)	Three samples of three titanium rigid UWC models	All wheelchairs passed the strength tests. Nine of 12 UWCs failed the MDT and CDT tests. Several backrest cane failures were noted. Sliding footrests and spoke failures on rear wheels were noted.

*Source*: Authors’ own work

ISO, International Organization for Standardization; MDT, multidrum test; CDT, curb-drop test; LRE, less-resourced environments; HWC, hospital style wheelchair; LWC, lightweight wheelchair; UWC, ultra-lightweight wheelchair.

### Wheelchair field failure evidence

**FIGURE 5:**
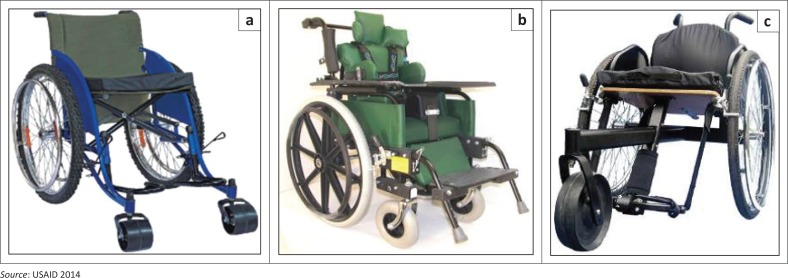
Wheelchair models designed for less-resourced environment use.

Failures found in field studies with different wheelchair models are listed in Table 2. Five of the reviewed studies (Armstrong et al. 2007; Reese & Rispin 2015; Rispin et al. 2012, 2013; Toro, Eke & Pearlman 2016) evaluated usability or durability aspects of wheelchairs designed for LREs. These models (see Figure 5) have passed ISO durability tests (Hof et al. 1993; USAID 2014) and were developed by non-profit organisations (Pearlman et al. 2008; Rispin et al. 2013). They are adjustable and more appropriate for rigorous use in rugged LRE conditions (Hotchkiss 1985; Sheldon & Jacobs 2006; Toro et al. 2016; Visagie et al. 2015a, 2015b, 2016). Despite wheelchairs passing ISO testing, breakdowns and failures occurred frequently and within months of the wheelchair being delivered, which reinforces the recommendation from the WHO guidelines that additional tests should be developed.

**TABLE 2:** Field failures of manual wheelchairs in less-resourced environments.

Author and year	Study details	ISO testing status	Maintenance status	Field failures
**Studies including HWC style designs**				
Toro et al. (2013)	Cross-sectional survey study conducted in a rehabilitation facility in Mexico. Paediatric users of donated HWCs (*n* = 43) were included in the study. Wheelchair use = 20 ± 16 months.	Wheelchairs failed on ISO test.	Self-repair and modifications	Failures noted were flat tyres and reattachment of drive wheel. This study reported extended results from an earlier study (Toro et al. 2012) reported below.
Shore and Juillerat (2012)	Cross-sectional survey study conducted in Vietnam, Chile and India. Donated semi-rigid HWCs (*n* = 519) were included in the study. Wheelchair use = 12 months.	Not ISO tested	Self-maintenance	A minimal repair rate of 3.3% was reported. Repairs were required for wheels, brakes, footrests and harness.
Toro et al. (2012)	Cross-sectional survey study conducted in a rehabilitation facility in Mexico. Paediatric users of donated HWCs (*n* = 23) were included in the study. Wheelchair use = 20 ± 16 months.	Not ISO tested	Self-repair and modifications to wheelchairs	Fifteen of 23 repairs or modifications were reported. Twenty of 23 wheelchairs were in damaged condition based on clinician rating. Inoperable brakes, loose seat and back-sling upholstery, worn out castors, cracked rear wheels and damaged armrests were reported.
Shore (2008)	Cross-sectional survey study conducted in Peru and India. Donated rigid HWCs (*n* = 188) were included in the study. Wheelchair use = 6–33 months.	Not ISO tested	Self-maintenance	Problems with flat rear tyres and tyre valves were reported. Minor issues with the resin chair were seen too. Twenty-eight per cent of users reported repairs within past 18 months.
Mukherjee and Samanta (2005)	Cross-sectional survey study conducted in India. Donated rigid HWCs (*n* = 162) were included in the study.	No data available on testing of the HWCs	No maintenance	Castors, wheel bearings, axles and solid tyres were reported to be frequently damaged. Extensive repair was required with very little wheelchair use. A total of 15.17% of wheelchairs were found to be damaged beyond repair.
Saha et al. (1990)	Cross-sectional survey study conducted in India. Locally produced HWCs (*n* = 50) from two manufacturers with wheelchair usage of 3–4 years.	No data available on testing of the HWCs	No maintenance	Multiple failures reported with castor bearings, fractures with spokes, footrests, castor wheels and forks. Brakes, seat and back material were found to wear rapidly. Rusted parts were observed.
**Studies with wheelchair models designed for LREs**				
Reese and Rispin (2015)	Cross-sectional survey study conducted in Kenya with paediatric users (*n* = 87). Failure data collected on five wheelchair models. Wheelchair use = 12–24 months.	Four of five wheelchair designs were ISO-qualified. The non-tested model was adapted from one of ISO-qualified model (Rispin & Wee 2014).	Irregular maintenance	Brakes were found to become loose, rusty or stiff and misadjusted. High occurrence of loose, wobbly hubs, some missing hand rims or nuts, worn tread and flat tyres were noted. Castors suffered from missing bearings and tyre cracking. Bent frames with rust and paint chips were observed. Armrests often showed significant degradation, breakage or loosening. Seats and seat backs showed collapsing of the foam. Their covers were cracked and torn. Common footrest problems were rotation stiffness, broken parts and obvious repairs, excessive looseness, cracked or broken foot plates, rusting and paint chips.
Rispin et al. (2012)	Cross-sectional study conducted in Kenya with paediatric users (*n* = 30). Failure data collected on two models: one model used for two weeks and the other one for eight months.	The model evaluated after two weeks of use was adapted from one of ISO-qualified model (Rispin & Wee 2014). The other model was ISO-qualified.	No maintenance	The ISO-qualified model had stiff brakes and broken trays and footrests. Some waterproof vinyl covers and cushions needed replacement. The other model had repeated flat tyres and misaligned wheels within two weeks of use.
**Studies with appropriate wheelchair provision of wheelchair models designed for LREs**				
Toro et al. (2016)	Paediatric and adult wheelchair users (*n* = 142) were evaluated in Indonesia. Four wheelchair models were provided. Wheelchair use = 6 months.	Two of four wheelchair designs were ISO-qualified	Self-maintenance	Fewer self-repairs with castors, seats, armrests, footrests, push handles and frames were reported overall.
Rispin et al. (2013)	Paediatric users (*n* = 10) in Kenya were evaluated following provision of two wheelchairs models. Wheelchairs were fit to users. Wheelchair use = 3 months.	ISO-qualified wheelchairs	No maintenance	Failures were noted with one chair only. Tyres were often flat. The seat and seat back fabric was more often cracked and torn. The cushions were collapsed. Manufacturing quality control issues were found with different parts.
Armstrong et al. (2007)	Prospective usability study (*n* = 100) conducted in Afghanistan with one wheelchair model. Three follow-up visits at weeks 3 and 10 and after 4 months were conducted. Failures reported are during the visits. Wheelchair use = 4 months.	ISO-qualified wheelchair	Self-maintenance, repairs and replacements conducted during follow-up visits by practitioners	Multiple brake handle issues and failures with seat fabric and rear wheel inner tubes were reported.

*Source*: Authors’ own work

ISO, International Organization for Standardization; HWC, hospital style wheelchair; LRE, less-resourced environments.

### Expert advice from International Society of Wheelchair Professionals Standards Working Group members

The ISWP-SWG members are co-authors of this article and their expertise is listed in Table 3.

**TABLE 3:** International Society of Wheelchair Professionals Standards Working Group member profiles.

Name	Professional position and current employer	Years of experience on wheelchairs	Work themes and topics of interest related to wheelchairs
Daniel Martin	Engineer, Shonaquip (South Africa)	7	Design and development of wheelchairs and posture support devices for use in LREs.
Matt McCambridge	Instructor, Research Engineer, Massachusetts Institute of Technology (United States)	16	Design, design facilitation, testing and manufacturing of mobility and posture support devices for use globally, training of technical staff involved in the manufacturing and distribution of mobility and posture support devices.
Norman Reese	Associate Professor, LeTourneau University (United States)	7	Test and design improvements for LREs.
Mark Sullivan (ISWP-SWG Chair)	Convaid (manufacturer of paediatric wheelchairs) and Polus Center (non-profit for prosthetics and wheelchair education and provision) (United States)	34	Product development of complex rehab wheelchairs for resourced countries, wheelchair seating education in LREs.
Don Schoendorfer	Founder, Free Wheelchair Mission (United States)	17	Providing mobility to the poor with disabilities in LREs.
Eric Wunderlich	Manager of Major Initiatives, LDS Church (United States)	12	Appropriate provision of wheelchairs in LREs.
David Mahilo	Director Corporate Reliability, Invacare (United States)	25	Wheelchair standards development, wheelchair testing, product development.
Chris Rushman	Technical Specialist, Motivation (United Kingdom)	22	Wheelchair product innovation, design and development, wheelchair production systems and production tooling design, wheelchair service training, technical training course or content design and development.
Anand Mhatre	Graduate Student Researcher, University of Pittsburgh (United States)	4	Wheelchair standards development, wheelchair testing, product development.
Jon Pearlman	Director, ISWP; Assistant Professor, University of Pittsburgh (United States)	15	Assistive technology transfer methods, design and development of products using participatory action design, wheelchair standards development and testing.

*Source*: Authors’ own work

ISWP-SWG, International Society of Wheelchair Professionals Standards Working Group; LRE, less-resourced environments.

**FIGURE 6:**
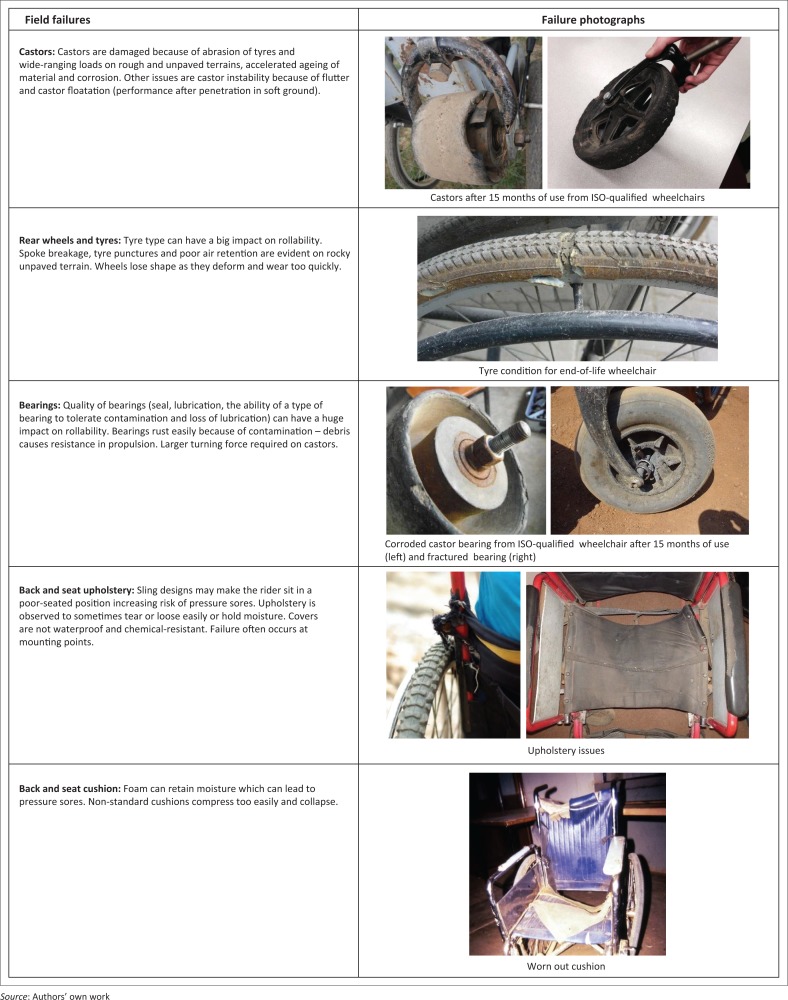

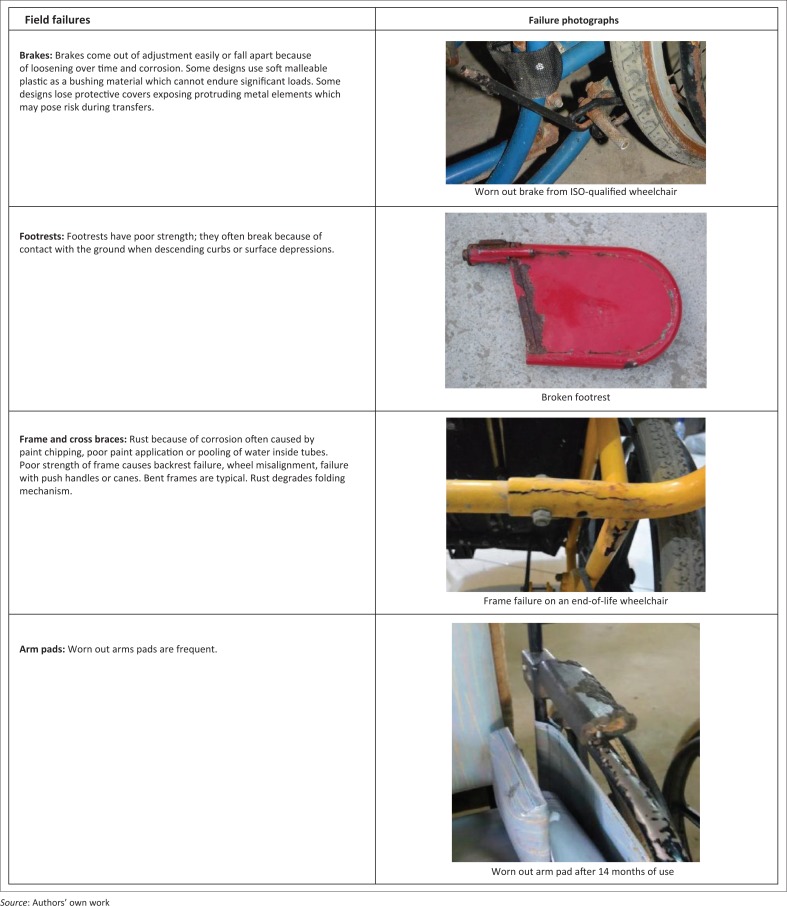
Failures noted by International Society of Wheelchair Professionals Standards Working Group experts on wheelchairs designed for less-resourced environment use.

International Society of Wheelchair Professionals Standards Working Group members reported minimal LRE participation in ISO 7176 standards development. They noted several product quality issues and failures seen typically in LREs as seen in Figure 6. Service delivery method and the status of maintenance, repairs and user skills training for wheelchairs in Figure 6 are unknown. These failures and breakdowns are irrespective of location of manufacture (locally produced or imported) and the context for use (REs or LREs). ISWP-SWG members identified certain unique quality-affecting elements such as corrosion, ageing and high impact forces (e.g. if a wheelchair is dropped from a bus) as causes for these failures. These elements are not present in ISO durability tests. Rapid breakdowns of components such as upholstery, anti-tippers, belt harness, calf straps, toe straps and fasteners were noted as durability issues that are not tested under ISO 7176.

### Additional test methods identification

To identify new tests, a product testing matrix as shown in Table 4 was put together that lists failure modes of different parts and the applicability of ISO test methods for predicting each failure mode. Testing priority was assigned by consensus from experts based on parts that fail most often and make the wheelchair non-functional. The lack of standard test methods (ISO, ASTM and MIL-SPEC) for predicting most failure modes on wheelchair parts led ISWP-SWG to identify new test methods.

**TABLE 4:** Product testing matrix.

Components	Failure modes	Test factors	Priority	ISO test methods
Castors, rear wheels and bearings	Tyre type, wheel and castor features and bearings affect rolling resistance	Rollability: effort required to propel wheelchairs on paved and unpaved surfaces	High	Not in ISO 7176
	Broken castor and wheel parts	Durability: impacts and loads; fracture loads		Yes (ISO 7176-8), but does not reproduce complex load conditions that occur in LREs
	Worn out tyres	Durability: abrasion		Not in ISO 7176
	Parts degradation	Durability: accelerated ageing		Not in ISO 7176
	Corroded bearings and metallic parts	Durability: corrosion		Not in ISO 7176
	Fluttering castor may waste effort and cause accidents	Castor flutter		Seen on ISO 7176-8 multi-drum test but not tested for
	Tyre puncture	Air retention for wheels, puncture tests		Not in ISO 7176
	Worn out bearings, dirt and dust in bearings	Test lubrication quality, seal design and quality		Not in ISO 7176
	Trueness of wheels over time is affected, camber issues	Wheel alignment		Not in ISO 7176
Seat cushion and upholstery	Seat cushions flatten over time	Durability: cushion compression	High	Not in ISO 7176
	Exposure to fluids causes deterioration	Chemical resistance and waterproof testing		Not in ISO 7176
	Tearing and wearing of cushion and cover, loosening upholstery	Durability: ageing, tearing, abrasion, loosening		Not in ISO 7176
Footrest	Broken footrests	Durability: strength	High	ISO 7176-8
	Difficulty in folding, adjusting for height	Durability: corrosion		Not in ISO 7176
Brakes	Loosening and corrosion of locking mechanism	Durability: cyclic testing, ageing, corrosion	Low	Not in ISO 7176
Frame and cross braces	Bent push handles	Durability: loading	Low	Not in ISO 7176
	Wear on coatings, coating deterioration	Paint chipping and corrosion		Not in ISO 7176
	Rusted holes, welds and areas where paint is chipped off	Durability: corrosion and testing folding mechanism		Not in ISO 7176
Fasteners and arm pads	Bolts and pads loosen out	Loosening	Low	ISO 7176-8
	Pads deteriorate, exposing edges	Ageing and abrasion testing		Not in ISO 7176
	Rusted components	Durability: corrosion		Not in ISO 7176

*Source*: Authors’ own work

ISO, International Organization for Standardization.

Castors and rear wheels were selected as crucial components that break down quickly in LREs and were prioritised for testing and test method development. Corrosion was identified as a variable that affects most wheelchair parts and was likewise prioritised for testing. Testing a complete wheelchair through simulated environmental conditions was considered as well.

## Discussion

Provision of appropriate, high-quality products is recommended in LREs as wheelchairs are subjected to adverse environments. Additionally, access to maintenance and repair services is limited in LREs. To ensure reliability of products, the WHO guidelines recommend conducting product testing based on ISO 7176 wheelchair standards. Current ISO test methods simulate conditions for urban paved environments and thus the development of additional test methods has been recommended for LREs based on typical conditions seen there (Borg & Khasnabis 2008). Following this recommendation, a prioritised list of tests was developed in this study through a literature review and feedback from experts to help predict wheelchair failures in LREs.

### Participation of less-resourced environments in standards development

As mentioned previously, there is little representation from LRE nations on the ISO testing committee, and consequently test methods do not completely reflect conditions in LREs. While WHO guidelines suggest using ISO 7176 as the basis to develop new standards for LREs (Borg & Khasnabis 2008), no new standards for this purpose have yet been proposed. Also, ISO standards testing has been carried out in resourced countries for more than 20 years for regulatory and research purposes, but no reports were found showing implementation of ISO 7176 in LREs thus far.

### Wheelchair durability

The ISO testing studies included in this review were conducted in an independent testing laboratory mostly on wheelchairs provided in REs. Results from Table 1 show that manual wheelchairs overall lack standard product quality, especially HWCs that resemble the majority of designs distributed in LREs (Constantine et al. 2006; Kim & Mulholland 1999; Pearlman et al. 2008; Zipfel et al. 2007). Around 70% – 90% of HWCs failed to pass minimum durability requirements (Cooper et al. 1996; Fitzgerald et al. 2001; Toro et al. 2013; Zipfel et al. 2007). Similar wheelchair designs produced in LREs (Toro et al. 2013; Zipfel et al. 2007) failed prematurely. Higher incidences of Class III failures with HWC designs indicate higher rates of breakdown and repairs during use, which is evident from anecdotal reports (Oderud 2014; Visagie et al. 2015a, 2015b) and reviewed field studies (Mukherjee & Samanta 2005; Saha et al. 1990; Toro et al. 2012, 2013). On the other hand, UWC designs were found to be durable and experienced fewer frame failures with ISO tests. This test outcome was predictable because UWCs are sophisticated wheelchair designs with superior quality materials that are designed for performance in developed environments and ISO durability tests subject wheelchairs to conditions that simulate such environments (Borg & Khasnabis 2008; Sheldon & Jacobs 2006). Field evidence with active users in REs has been reported with UWCs which shows positive satisfaction and fewer repairs in last six months of use (Fitzgerald et al. 2005). However, these designs are not suitable for LREs owing to high costs associated with their materials and manufacturing. Overall, it can be concluded that ISO durability tests are suitable to test wheelchair designs like HWCs that break prematurely and UWCs that are developed for performance in REs.

### Durability of International Organization for Standardization-qualified models

Field evaluation studies have been carried out with ISO-qualified wheelchairs appropriate for LREs. Four such field studies reported failures, repairs, replacements and missing parts over two weeks to eight months of field use (Armstrong et al. 2007; Rispin et al. 2012, 2013; Toro et al. 2016). Wheelchairs in two of these short-term studies were provided based on WHO guidelines (Borg & Khasnabis 2008; World Health Organization 2012, 2013), maintained frequently and favoured by the users (Armstrong et al. 2007; Toro et al. 2016). One study (Reese & Rispin 2015) assessed ISO-qualified appropriate wheelchairs after 1–2 years of use which were provided without user training and serviced occasionally. Several part failures were found that would require a technician’s attention (see Table 2). Findings from these studies demonstrate that failures occur on ISO-qualified models with everyday use especially with parts such as brakes, tyres, seat covers, castors, footrests and armrests. Field failures can be associated with product properties such as substandard material quality, poor parts selection, inappropriate design and manufacturing inconsistencies. These properties can vary with the locally produced versions of certain ISO-qualified wheelchairs like the Whirlwind Roughrider which makes them prone to early failure. Moreover, LRE environments are harsh and can degrade products rapidly. ISO test qualification is representative of 3–5 years of outdoor use (Cooper 1998; Hobson 1999) but apparently falls short of qualifying products for LRE use based on reviewed study results. Accurate prediction of life duration of certain wheelchair parts may not be guaranteed.

### Wheelchair failures

Failures seen with ISO testing in the laboratory were similar among wheelchair designs. Fractures with cross braces, side frames (at weld joints), backrests, castor spindles and footrests were found to be common in these studies. Failures were influenced by frame design, wheelchair material, screw holes, welding techniques and castor and tyre characteristics. However, failure modes observed with ISO testing are rare in the field based on field failure evidence gathered through literature review and failure evidence provided by ISWP-SWG members (see Figure 4). Dominant field failures found in LREs are flat and cracked tyres, wobbly rear wheels, bent frames, non-functional brakes, worn out bearings, damaged armrests, torn seat covers, loose upholstery, collapsed cushions and rusting and loosening of several parts. Any representation of these failures is not evident in ISO testing results which mostly produces fracture failures caused by impacts on MDT and CDT. This is because test conditions employed on ISO durability tests do not simulate environmental and demanding use conditions from LREs. To accurately predict failure modes and life duration of products for LREs, it is necessary to develop additional testing methods for LREs with relevant test conditions. ISWP-SWG experts echoed similar advice.

### Identification of additional test methods

The product testing matrix developed through consensus of experts highlights the requisite test factors (conditions) for testing products for LREs. The matrix assisted in development of additional test methods for LREs. Based on availability of resources and capacity for development with ISWP partners in the SWG, four test methods were given high priority – castor durability testing, rolling resistance testing, corrosion testing and whole chair testing.

Castor failure was noted as a top concern in the field as per ISWP-SWG experts. Castors experience a variety of failure modes with tyres, bearings and stem hubs and ISO tests primarily subject castors to vertical loads and stresses. Hence, experts suggested that castor durability testing should be conducted separately. Incorporating amplified and angular loading patterns along with corrosive conditions and various types of simulated surfaces including sand, mud, gravel and stones is recommended for the new castor test method. Such testing is estimated to screen castor designs for greater durability, requiring less maintenance and incurring fewer repairs in LREs.

Corrosion of wheelchairs was observed as a critical concern because several wheelchair components are unable to operate after being rusted. Although ISO testing includes climatic testing of wheelchairs in hot and cold environments for power wheelchairs only, it does not simulate moisture and acidic exposure. It is known that corrosion adds to the effect of fatigue during field use for certain wheelchair parts like bearings. This calls for conducting fatigue and corrosion testing simultaneously. Experts recommended corrosion evaluation of the complete wheelchair similar to already established standards like ASTM B117.

Resistance to wheelchair rolling was also identified as a major performance issue in LREs. While resistance characteristics for rubber on different surfaces are known to an extent, propelling wheelchairs over a variety of surfaces requires a significant user effort (Armstrong et al. 2007; Oderud 2014; Rispin & Wee 2014). Wheels experience a range of rolling resistances based on variation of elastic rebound between the tyre and different surfaces, tyre tread design, type of tyre (pneumatic vs solid), camber angle, toe-in and toe-out alignment, type of spokes and characteristics of the axle hub bearings. Castors are also known to have greater rolling resistances based on tyre diameter, characteristics, surface, the type of materials used and bearing efficiency. Thus, testing to evaluate the rolling of wheels and castors, which is not a part of ISO 7176, is being considered in the new test methods. Comparing the rolling resistance of different types of available wheels and castors is critical so that performance products can be selected.

The ISWP-SWG recognises that the entire wheelchair suffers from different types of loads and effects of environmental factors causing wear (ultraviolet light, high temperature, dirt and dust) and corrosion (humidity and water exposure). None of the ISO tests subject wheelchairs to a combined effect of these test factors. Hence, testing the complete wheelchair simultaneously against relevant test factors to replicate failures as seen in the field is suggested.

### Observations from field studies

Field studies that provided wheelchairs as per WHO guidelines (Armstrong et al. 2007; Toro et al. 2016) indicated that appropriate services, user training and regular maintenance are necessary to reduce the rate of field failures. However, LREs struggle with capacity for appropriate services. Provision of user training, funding and access to repair services is limited (Armstrong et al. 2007; Borg & Khasnabis 2008; Hof et al. 1993; Oderud 2014; Sheldon & Jacobs 2006; Visagie et al. 2013, 2015b) which was evident in field studies as well (Mukherjee & Samanta 2005; Reese & Rispin 2015; Rispin et al. 2012, 2013; Saha et al. 1990). In the wake of such concerns, international efforts focused on increasing capacity and improving service provision in LREs are ongoing (International Society of Wheelchair Professionals 2015). While such efforts are in progress, it is equally necessary to develop products with greater reliability and higher durability to reduce failure occurrences and prevent breakdowns. This perspective has been shared by the WHO guidelines as well which stress the parallel need for appropriate services and high-quality products (Borg & Khasnabis 2008; United Nations 2006). Development of durable, high-quality products, in turn, calls for development of rigorous test methods which were identified in this study.

## Limitations

This study is limited by the variable methodologies and availability of evidence. The study pulls evidence from ISO testing studies and field evaluation studies combined with expert recommendations to determine additional product quality tests. Based on the review, a low level of evidence for products used in LREs is available to inform additional test development for LREs. Twelve research articles were included in this literature review on wheelchair testing and only two studies reported results with wheelchairs used in LREs. Although the USAID report (USAID 2014) on wheelchairs recommends ISO testing of wheelchair designs appropriate for LREs, full-fledged ISO testing studies with such designs are not yet conducted. Findings from such studies could have assisted in understanding the failures in the laboratory and directed the additional test development.

Field evidence in the review was limited in many respects. Numbers of failures, repairs and replacements were provided in four studies out of which two were conducted in a rehabilitation facility and two evaluated HWCs (Armstrong et al. 2007; Shore 2008; Shore & Juillerat 2012; Toro et al. 2012, 2013, 2016). Otherwise, only different failure types were reported. There was a lack of evidence on whether failures led to breakdowns (usually caused by severe damage to frame, castor or rear wheel) except for one study on donated wheelchairs (Mukherjee & Samanta 2005). Several studies involved modifications to the products prior to evaluations which could have affected the failure outcomes (Reese & Rispin 2015; Toro et al. 2012). Nearly all studies with ISO-qualified appropriate models (Reese & Rispin 2015; Rispin et al. 2013; Toro et al. 2012, 2013, 2016) involved paediatric populations whose functional requirements from a wheelchair, use practices, hours of use per day, method of propulsion and maintenance abilities are different from the general population. In a broader population of adults, it is expected that failures would be quicker and more severe with the same designs. There were no long-term studies which could have allowed for better comparison with ISO tests. Also, no comparison studies were found between REs and LREs.

Among the ISWP-SWG experts, only one comes from LRE. As expert advice was sought in this study, there is a potential for expert bias in this study. Photographs collected as evidence were only available from end-of-life chairs which may indicate extreme damage to the part, with limited knowledge of the age or conditions of use of the chair.

## Future work

Following development of test methods, the ISWP-SWG group plans to suggest new test methods to the ISO standards committee as a new or add-on standard to ISO 7176 or as a technical specification so that they are harmonised with national standards. Product quality testing using these additional standards could then be included as part of regulatory policies that governments of less-resourced countries adopt, or as part of the WHO GATE initiative. These standards can support implementation of the UN-CRPD Article 20, which many countries around the world have agreed to adopt. Validation of the new test methods is an important step to assess correlation with performance seen in the field, and will be conducted through research studies in LREs in collaboration with manufacturers and charitable organisations. Manufacturers and wheelchair designers in LREs will be encouraged to implement ISWP test methods for testing newly designed parts, custom components and wheelchair prototypes. Parts with low testing priority will be tested as resources are available.

## Conclusion

The goals of this work were to identify the additional wheelchair tests necessary to screen products for LREs as suggested by the WHO guidelines and Wheelchair Consensus Conference (Sheldon & Jacobs 2006; Borg & Khasnabis 2008). Through literature search and review, several studies were found that tested products on ISO 7176 laboratory tests and in LREs. However, none investigated testing products for LRE-like conditions in laboratory settings. This is the first study addressing developing standard test methods for LREs. Published evidence combined with field observations by ISWP-SWG experts indicates that wheelchairs fail in LREs in ways that would not be predicted by ISO 7176 tests. Additional test methods are required that incorporate test factors responsible for the diverse failures seen in LREs. The additional tests that were identified include testing for castor durability, corrosion because of the environmental conditions, rear wheel and castor rolling resistance and whole chair durability. The ISWP-SWG is in the process of developing and validating these additional test methods. Results from such testing can assist manufacturers and designers in understanding deficiencies in materials and in discovering design flaws that make the product unsuitable for LRE conditions. Additionally, information from test results can inform providers and clinicians in LREs regarding durability and reliability of products which can guide wheelchair selection and delivery.
